# Editorial: Neuroengineering for health and disease: a multi-scale approach

**DOI:** 10.3389/fnins.2026.1929592

**Published:** 2026-07-29

**Authors:** Michela Chiappalone, Gabriele Arnulfo, Paolo Massobrio, Marco Fato, Alberto Mazzoni, Sergio Martinoia

**Affiliations:** 1 Neuroengineering Genoa Group, Department of Informatics, Bioengineering, Robotics, and Systems Engineering—DIBRIS, University of Genova, Genova, Italy; 2 Life Science and Technologies Lab—LisTechLab, AOM - IRCCS Ospedale Policlinico San Martino, Genova, Italy; 3 Laboratorio di Medicina Computazionale e Tecnologica, IRCCS Istituto Giannina Gaslini, Genova, Italy; 4 Computational Neuroengineering Laboratory, Istituto di Biorobotica, Scuola Superiore Sant'Anna, Pisa, Italy

**Keywords:** artificial intelligence—AI, computational modeling, multi-scale, neural signal analysis, neuroengineering

The brain is inherently multi-scale, characterized by complex interactions that span from molecular and cellular levels to entire networks and behaviors (Bassett and Sporns, 2017). This complexity presents significant challenges for understanding brain function in both health and disease and requires approaches capable of analyzing and integrating information across multiple spatial and temporal scales.

Neuroengineering has emerged as a key interdisciplinary field to address these challenges by combining principles from neuroscience, engineering, computational modeling, and advanced technologies (D'Angelo and Jirsa, 2022; Xiong et al., 2023). By integrating experimental and computational approaches, neuroengineering contributes to a more comprehensive understanding of neural dynamics across different levels of biological organization. In parallel, it also drives the development of technologies capable of interacting with the nervous system, including brain–machine interfaces (BMIs), neuroprostheses, and targeted neuromodulation strategies, with the ultimate goal of improving the diagnosis, treatment, and rehabilitation of neurological disorders (Carè et al., 2024; Chiappalone et al., 2022; Lebedev and Nicolelis, 2017; Tonin et al., 2025).

Reflecting this broad perspective, this Research Topic brings together contributions illustrating the multidisciplinary nature of contemporary neuroengineering, from understanding neural function to developing technologies capable of interacting with and restoring the nervous system. The published studies span a broad range of methodologies, including advanced neuroimaging, electrophysiology, artificial intelligence (AI), computational modeling, neural interfaces, and neuromodulation. Together, these contributions illustrate how neuroengineering integrates complementary methodologies and experimental models across multiple scales, providing the conceptual basis for the thematic perspectives discussed in this Editorial.

The Research Topic originated from the scientific discussions that took place during the 9th edition of the *Summer School on Neuroengineering “Massimo Grattarola”* (https://neuroengineering.eu), which was held in Genova, Italy in June 2024. The Summer School, held every 2 years, brought together PhD students, young researchers, clinicians, engineers, and internationally recognized scientists working across the many facets of neuroengineering, creating a stimulating environment for scientific exchange and interdisciplinary interaction. Named after Massimo Grattarola (1950–2002, https://en.wikipedia.org/wiki/Massimo_Grattarola), one of the pioneers of bioengineering and bioelectronics in Italy, the School reflects his vision of integrating engineering, physics, biology, and medicine to better understand biological systems and develop innovative biomedical technologies. His scientific legacy continues to inspire research at the interface between neuroscience and engineering.

The Research Topic hosts 13 articles, including seven Original Research articles, two Mini Reviews, two Perspectives, and two Case Reports. For the purpose of this Editorial, the published contributions can be viewed through three complementary perspectives, i.e., *Reading, Writing*, and *Restoring the brain*. Rather than representing distinct domains, these perspectives capture complementary dimensions of multi-scale neuroengineering. Together, they reflect the different yet interconnected ways in which neuroengineering contributes to understanding, interacting with, and ultimately restoring nervous system function. While these themes are not intended as rigid categories (indeed, several contributions naturally span more than one) they provide a useful framework for appreciating the breadth of the Research Topic and the increasingly integrated nature of neuroengineering.

The theme “*Reading the brain*” brings together studies aimed at extracting meaningful information from the nervous system through advanced neuroimaging, electrophysiology, computational modeling, and AI, in different experimental models, from *in vitro* to *in vivo* and human studies. Despite addressing different neurological conditions and methodological approaches, these contributions share the common objective of decoding neural function across multiple biological scales to improve disease characterization, biomarker discovery, and data-driven clinical decision-making. Grasselli et al. provide a comprehensive overview of the electrophysiological characterization of *in vitro* models of Parkinson's disease. The authors discuss the opportunities offered by techniques such as patch-clamp recordings and microelectrode arrays to investigate neuronal dysfunction and network alterations, while highlighting the need for standardized experimental protocols to improve reproducibility and accelerate biomarker discovery. Canu et al. investigate how ischemic lesions reshape functional connectivity within the sensorimotor network during sleep in rodent models *in vivo*. Through the analysis of local field potentials, phase synchronization, and cross-frequency coupling, the study identifies transient changes associated with post-stroke plasticity, suggesting potential electrophysiological biomarkers that could guide future neurorehabilitation and neuromodulation strategies. Wu et al. introduce DSCNet, a deep-learning framework designed to identify neural signatures of drug and alcohol addiction from EEG recordings. By combining multi-angle feature extraction with adaptive feature modulation, the proposed approach significantly improves classification performance, highlighting the growing role of artificial intelligence in decoding complex brain signals and supporting objective diagnostic strategies. Trò et al. propose an innovative framework for integrating multiple advanced diffusion MRI microstructural models to investigate brain alterations associated with preterm birth. By combining complementary imaging biomarkers through both inferential and predictive approaches, the study demonstrates how multimodal neuroimaging can provide a more comprehensive characterization of white matter development and improve the identification of subtle neurodevelopmental alterations. Jiang et al. present a predictive framework for assessing the risk of acute ischemic stroke in patients with non-valvular atrial fibrillation by integrating heterogeneous sources of information, including clinical variables, cardiac anatomical and functional features, electrophysiological findings, hemodynamic parameters, and circulating biomarkers. Their work highlights the growing importance of multimodal data integration to support personalized risk stratification and clinical decision-making.

The theme “*Writing the Brain*” focuses on technologies that actively interact with the nervous system through electrical stimulation and neuromodulation, opening new opportunities for targeted and personalized interventions. The related studies illustrate how advances in stimulation paradigms, neural interfaces, and neuromodulation strategies are progressively moving from mechanistic investigations toward clinically relevant applications, highlighting the growing ability of neuroengineering not only to record neural activity, but also to shape it. Asamoah et al. investigate the neuronal effects of epicranial current stimulation in non-human primates, positioning this emerging approach as an intermediate solution between non-invasive transcranial stimulation and invasive cortical stimulation. By combining electrophysiological recordings with functional imaging, the study provides compelling evidence of the neuromodulatory potential of epicranial stimulation and supports its future translation into clinical applications. Leukhin et al. propose a novel paired associative peripheral nerve stimulation paradigm that selectively modulates neural activity by exploiting differences in axonal conduction velocities. Tested in healthy volunteers, the approach demonstrates the feasibility of inducing controlled inhibitory motor responses and represents a promising step toward personalized, non-invasive neuromodulation strategies. Deng et al. present a case series describing the successful use of transcutaneous facial nerve stimulation for the treatment of intractable postherpetic neuralgia in patients with typical and atypical Ramsay Hunt syndrome. Although preliminary, the results demonstrate the therapeutic potential of targeted peripheral neuromodulation for managing otherwise difficult-to-treat neuropathic pain conditions. Francisco Silvestre et al. report two clinical cases investigating the long-term effects of neuromuscular electrical stimulation in patients with spinal cord injury. The observed improvements in neurological status and muscle activation suggest that electrical stimulation can promote neuroplasticity and functional recovery, further supporting its role within rehabilitation protocols. Finally, Tecchio and Paulon broaden the perspective by discussing the role of non-invasive neuromodulation in alleviating chronic fatigue across neurological disorders. Integrating current clinical evidence with neurophysiological mechanisms, the authors propose neuromodulation as a key component of personalized, multidisciplinary therapeutic strategies aimed at restoring sensorimotor balance and improving quality of life.

The theme “*Restoring the brain*” highlights the translational nature of neuroengineering, demonstrating how advances in this field can converge toward clinically relevant solutions. The contributions in this section illustrate different but complementary strategies for restoring lost neurological function, ranging from the identification of novel therapeutic targets to the development of advanced human–machine interfaces and bio-inspired control systems, thereby emphasizing the continuum from mechanistic understanding to functional recovery. Kilic et al. investigate the electrophysiological properties of abnormal brain cavities that develop following ischemic stroke, demonstrating that the cavity walls retain organized neural activity and may represent promising targets for future neuromodulation therapies. By characterizing local field potentials, event-related potentials, and spiking activity, the study provides new insights into post-stroke plasticity and identifies novel opportunities for targeted functional restoration. Quadrelli et al. review recent advances in high-density electromyography (HD-EMG) interfaces and spatial machine-learning algorithms for upper-limb prosthetic control. By discussing both technological developments and current translational challenges, the authors highlight how increasingly intuitive human-machine interfaces may improve prosthesis control and contribute to restoring motor function in individuals with limb loss. Perricone et al. propose a bio-inspired computational framework for the physiological control of artificial respiration based on neuronal models of the respiratory central pattern generator. Their perspective illustrates how computational neuroscience and closed-loop control strategies may pave the way toward more adaptive and physiologically meaningful respiratory support systems, ultimately improving patient care during assisted ventilation.

The *Reading, Writing*, and *Restoring the brain* perspectives illustrated in this Editorial offer an integrated view of contemporary multi-scale neuroengineering. Although the contributions address diverse scientific questions and span different levels of biological organization, experimental models, and technological approaches, they converge toward a common objective: understanding nervous system function, interacting with neural circuits in a controlled manner, and ultimately translating these advances into clinically relevant solutions. This broader view reflects the increasingly close interplay between fundamental neuroscience, engineering, and clinical research that is driving the evolution of modern neuroengineering. Overall, this Research Topic gave both the authors and the Guest Editors the opportunity to contribute to a shared vision of neuroengineering, where reading, writing, and restoring nervous system function across scales and in different experimental models represent complementary steps toward more effective and personalized healthcare. We hope that this Research Topic will inspire new interdisciplinary collaborations and further advance the translation of neuroengineering research from laboratory to clinical practice.
